# Effectiveness of Reverse Sural Artery Flap in the Management of Wheel Spoke Injuries of the Heel

**DOI:** 10.7759/cureus.1331

**Published:** 2017-06-10

**Authors:** Hafiz U. Farooq, Rizwan Ishtiaq, Shabana Mehr, Sadia Ayub, Umer H Chaudhry, Anam Ashraf

**Affiliations:** 1 Department of Plastic Surgery, Bahawal Victoria Hospital Bahawalpur; 2 Gastroenterology, Beth Israel Deaconess Medical Center, Boston; 3 Department of General Surgery, Bahawal Victoria Hospital Bahawalpur; 4 Internal Medicine, Rawalpindi Medical College, Rawalpindi, Pakistan

**Keywords:** reverse sural artery flap, wheel spoke injury, heel

## Abstract

**Objective:**

Soft tissue injuries at the level of lower extremities, plantar, and dorsal foot pose a surgical challenge for reconstructive surgeons. This kind of injury commonly occurs when lower limbs get stuck in between the spokes of the wheel. Reverse sural artery flap has been proven to be an effective option to cover such defects. The aim of this study is to analyze the demographic variables of affected individuals, technical aspects of reverse sural artery flap, quantify the effectiveness of reverse sural artery flap among various treatment options available, and to study the outcome of injury.

**Methods:**

A total of 49 patients who presented during a period of six years from January 2010 to January 2016 were included in the study. The data was collected using patient’s charts, by interviewing the patients, and from hospital records. The patients' wounds were prepared, examined, and the injury was graded depending upon the extent of tissue damage. Tendon and bone defects were repaired, and wounds were closed by either split thickness skin graft or reverse sural artery flaps.

**Results:**

Children were the most commonly affected with no conclusive gender trend. The posterolateral part of the heel of the right foot was the most frequently injured part (69%). Surgical interventions together with proper postoperative care and follow-up produced very good results overall.

**Conclusion:**

Wheel spoke injuries of the heel can be managed without significant morbidity if the patient presents early, the wound is assessed properly, suitable surgical technique is utilized, and good postoperative care is provided.

## Introduction

Wheel spoke injuries occur when the feet of passengers get trapped in the rotating spokes of the wheels of a bi-wheeler. Reports of these injuries have continuously been appearing in the literature since these were reported for the first time in 1948 [1]. These types of injuries are quite common, especially in urban and suburban regions of developing countries like Pakistan where motorbikes are becoming an increasingly common mode of transport. Children are prone to be affected due to their small-sized feet, which passes easily through the spokes of the wheels of the vehicles [2]. The common causes of such accidents are reported to be overloading, inappropriate footwear, and absence of spoke guards and foot rests. Most passengers suffer right-sided foot injury due to the left-sided placement of chain guards that prevent probable entanglement [3]. The heel is the commonly affected part, which being the most pressure bearing, poses multiple problems to the patients and to the healthcare providers as the duration of healing is usually prolonged and sometimes multiple surgeries are needed. The spectrum of injury ranges from soft tissue lacerations to crushing and subtotal amputation of the foot and ankle. A grading system that determines the extent of tissue damage and the treatment modality is shown in Table 1.

**Table 1:** Grades of wheel spoke injury based on severity of injury and tissue damage.

Grade	Wound description	Management employed
1	Skin loss with no exposure of bone or tendon	Split thickness skin grafting
2	Skin loss with Achilles tendon either exposed or ruptured	Achilles tendon repair and reverse sural artery flap
3	Skin loss with Achilles tendon defect, calcaneus exposed or fractured	Management of fracture using wires and screws, Achilles tendon transfer and reverse sural artery flap
4	Mangled foot with damage to neurovascular bundles	Amputation

## Materials and methods

A prospective study was designed to study a total of 49 patients admitted in the department of plastic surgery at the Combined Military Hospital, Lahore and Bahawal Victoria Hospital, Bahawalpur during a period of six years from January 2010 to January 2016. Data was gathered with the help of patient’s charts, interviews of the patients, and from hospital records. It included age, sex, foot affected, total hospital stay, treatment implicated, and outcomes. The patients' wounds were prepared, examined, and the injury was graded depending upon the extent of tissue damage.

**Figure 1:**
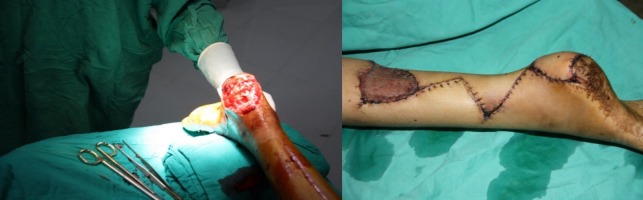
Pre-operative (left) and post-operative (right) picture of the left foot of a 15-year-old girl, which was repaired with reverse sural artery flap.

**Figure 2:**
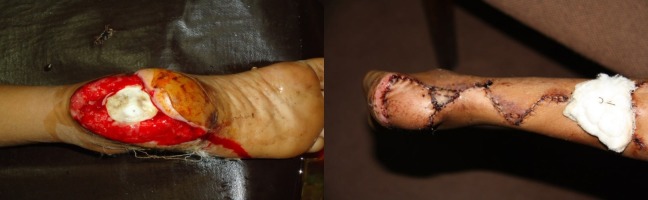
Pre-operative (left) and post-operative (right) picture of the foot of a 14-year-old boy after wheel spoke injury.

Repeated debridement and dressings of the wounds were performed until the wound was ready for further management. X-ray scans were performed regularly to rule out osteomyelitis. Split thickness skin grafts, harvested from the thigh of the unaffected limb were used to cover grade 1 wounds. Ruptured tendons in grade 2 injuries were repaired primarily using proline 4.0 or 5.0 sutures and the skin defect was closed by reverse sural artery flap as shown in Figures 1-2.

**Figure 3:**
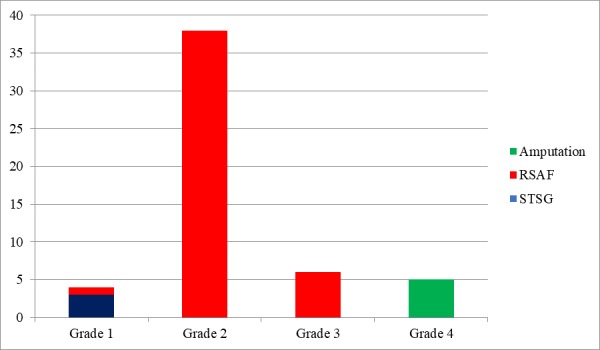
Graphical representation of different surgical interventions utilized for the coverage of wheel spoke injury of the heel. RSAF - Reverse sural artery flap, STSG - Split thickness skin graft.

Plaster of Paris was applied to immobilize the limb. Calcaneal fractures were managed by the orthopedic department with the help of wires and screws. Achilles tendon defects were repaired by flexor hallucis longus tendon transfer. The wound was closed and the limb immobilized in the same way as in the case of grade 2 injuries. Amputation was the treatment of choice in the case of grade 4 injuries as there was no hope of foot salvage. Figure 3 gives an overview of the surgical intervention depending upon the grade of the injury.

Vascularity of the flap was regularly monitored by observing its color and pin pricks. In the case of peripheral flap necrosis, the necrotic area was debrided and the wound was closed by secondary intention afterward. Patients were evaluated for the functionality of foot on their follow-up visits three months postoperatively.

## Results

The mean interval from injury to presentation was seven (2-14) days. Thirty-four cases were reported with right foot injury and 15 cases were reported with left foot injury. The hind foot was the site of involvement in all cases. The different variables of injury along with the mode of treatment are listed in Table 2.

**Table 2:** Variables of wheel spoke injuries in our study. RSAF – Reverse sural artery flap, STSG – Split thickness skin graft.

Grades	Number of Cases	Average Age (Years)	Sex (M/F)	Foot involved (R/L)	Treatment	Hospital Stay (Days)
1	4	7	2/2	2/2	STSG	7
2	38	12	22/17	11/27	RSAF	12
3	6	11	3/3	2/4	RSAF	14
4	1	16	0/1	0/1	Amputation	15

In three out of four cases of grade 1 injuries, grafts were taken up successfully. Postoperative infection led to graft loss in the fourth case, which was later treated by reverse sural artery flap. Seventy-seven percent (n=38) of cases were categorized as grade 2. Achilles tendon rupture was seen in only one case, which was repaired as mentioned earlier.

Twelve percent (n=6) of the patients had grade 3 injury. Calcaneus fractures and Achilles tendon loss was seen in two cases. Reverse sural artery flap was done in 44 patients who suffered grade 2 and grade 3 injuries. Ninety-three percent (n=41) of the flaps were taken up completely. Peripheral flap necrosis was observed in three wounds. The average hospital stay of patients with grade 1, 2, 3 and 4 injury was seven, 12, 14, and 15 days, respectively.

Patients were evaluated three months postoperatively and were asked questions regarding their ability to fulfill daily life activities. All patients were able to resume their daily life activities.

## Discussion

The demographic variables of wheel spoke injury in our study are in consistency with those reported in previous literature [4-8]. The severity of the injury is directly related to the speed of the vehicle, as seen with motorcycle injuries, which are quite deeper and are of higher grades. All grade 1 injuries were caused by bicycles. The grading system employed in our study is a bit different from previous studies done in Pakistan, India, and China [4-5]. We think that this grading system suits our setting better as far as management is concerned. In a study conducted in the Netherlands, the role of HydroBalance dressings (Lohmann & Rauscher, Germany) was evaluated for the management of grade 1 and 2 injuries [9]. Although these dressings may be quite effective, they are of limited value in our setup considering their cost and availability. Reverse sural artery flap is the only treatment employed by us for the coverage of deeper grade 2 and 3 injuries in contrast to other authors who did various types of distant and local flaps and microvascular tissue transfer too, which required more resources and expertise [4-5]. The results are comparable in these cases indicating the importance of reverse sural artery flap, which is a good option in settings with limited resources and in operation theaters that are not equipped with microvascular tissue transfer facilities. Akhter S and Hameed A utilized sural fasciocutaneous flap for the coverage of wounds of the lower leg and ankle due to different etiologies including trauma, wheel spoke injury, trophic ulcers, osteomyelitis, Marjolin's ulcer, and diabetic ulcers [10]. The proportion of flap complications is higher in their study, which may be because of patients with relatively older age groups and comorbid conditions like diabetes [11-12].

## Conclusions

According to our study, 93% (n=41) of the patients had their flaps taken up completely without any complications. Almost all the patients could continue their daily activities three months after the surgery. This shows that reverse sural artery flap is an effective option for the coverage of heel injuries in wheel spoke accidents. It can be quickly performed and microsurgical skills are not required.
